# Clinical features of patients with hepatic portal venous gas

**DOI:** 10.1186/s12893-020-00973-8

**Published:** 2020-11-27

**Authors:** Manato Fujii, Suguru Yamashita, Mayuko Tanaka, Jo Tashiro, Yoshiharu Takenaka, Kazuki Yamasaki, Yukiyoshi Masaki

**Affiliations:** 1 Department of Surgery, Ome Municipal General Hospital, 4-16-5, Higashi Ome, Ome-shi, Tokyo, 198-0042 Japan; 2 Department of Radiology, Ome Municipal General Hospital, 4-16-5, Higashi Ome, Ome-shi, Tokyo, 198-0042 Japan; 3 Department of Surgery, St. Luke’s International Hospital, 9-1 Akashicho, Chuo-ku, Tokyo, 104-8560 Japan

**Keywords:** Hepatic portal venous gas, Bowel ischemia, Predictive factors

## Abstract

**Background:**

Hepatic portal venous gas (HPVG) is a rare clinical condition that is caused by a variety of underlying diseases. However, the factors that would permit accurate identification of bowel ischemia, requiring surgery, in patients with HPVG have not been fully investigated.

**Methods:**

Thirty patients that had been diagnosed with HPVG using computed tomography between 2010 and 2019 were allocated to two groups on the basis of clinical and intraoperative findings: those with (Group 1; n = 12 [40%]) and without (Group 2; n = 18 [60%]) bowel ischemia. Eleven patients underwent emergency surgery, and bowel ischemia was identified in eight of these (73%). Four patients in Group 1 were diagnosed with bowel ischemia, but treated palliatively because of their general condition. We compared the characteristics and outcomes of Groups 1 and 2 and identified possible prognostic factors for bowel ischemia.

**Results:**

At admission, patients in Group 1 more commonly showed the peritoneal irritation sign, had lower base excess, higher lactate, and higher C-reactive protein, and more frequently had comorbid intestinal pneumatosis. Of the eight bowel ischemia surgery patients, four (50%) died, mainly because of anastomotic leak following bowel resection and primary anastomosis (3/4, 75%). All except one patient in Group 2, who presented with aspiration pneumonia, responded better to treatment.

**Conclusions:**

Earlier identification and grading of bowel ischemia according to the findings at admission should benefit patients with HPVG by reducing the incidence of unnecessary surgery and increasing the use of safer procedures, such as prophylactic stoma placement.

## Background

Hepatic portal venous gas (HPVG), which was first recognized in neonates by Wolf et al. [1] and in adults by Susman et al. [2], has been reported to be associated with three major inciting factors: bowel mucosal damage, bowel distention, and sepsis [3]. On the basis of early studies that used plain abdominal radiography, HPVG was regarded as a life-threatening pathology that principally reflected bowel ischemia [3, 4]. However, the advent of more advanced imaging techniques, such as multi-detector computed tomography (CT), which have excellent spatial and contrast resolution, have increased the sensitivity of HPVG detection [5, 6], and consequently the number of reports of HPVG patients without bowel ischemia. Specifically, HPVG that is not related to abdominal pathology [7, 8] and iatrogenic HPVG [9–14] are increasingly recognized in this era of multi-disciplinary examinations and interventions. Then, the more recent literature [15, 16] has reported a reduction in the mortality rate of patients with HPVG to ~ 40%, due to the study of larger number of patients that did not have bowel ischemia, which compares with the oft-cited mortality rates described in earlier reports of ~ 75% [3].

HPVG is not a specific disease, but instead can be attributable to a variety of contrasting underlying pathologies. Since bowel ischemia has been widely recognized as the leading lethal etiological factor among many background pathologies of HPVG [17], it is clinically essential to identify the presence of bowel ischemia and to grade its severity in HPVG patients. Although there is a growing body of literature regarding HPVG patients who did not have life-threatening pathology, in the context of the advances in imaging sensitivity, HPVG remains a rare condition and the majority of the literature comprises case series. Indeed, recent researchers have calculated the overall incidence of HPVG to be only 0.06%–0.12%, on the basis of retrospective radiologic reviews conducted in academic medical settings [18, 19]. As such, the factors involved in the accurate prediction of bowel ischemia in patients with HPVG have not been able to be fully investigated to date.

The aims of the current study were two-fold: (1) to report the characteristics and outcomes of a recent set of consecutive HPVG patients at a tertiary hospital comparing those with data in the published article, and (2) to determine the predictors for the identification of bowel ischemia in such a cohort.

## Methods

### Study design and population

This retrospective study was performed at a single tertiary hospital in Tokyo, Japan. The study protocol conformed to the ethical guidelines of the Ome Municipal General Hospital, as reflected in a priori approval by the institutional review board (Referee number, 46). Because of the retrospective nature of the study, informed consent was not required. All procedures were conducted in accordance with the principles of the Helsinki Declaration of 1975, as revised in 1983.

Between 2010 and 2019, we enrolled 30 consecutive adult patients with HPVG diagnosed using multi-detector CT (Aquilion 64, Canon, Japan) by the experienced attending diagnostic radiologist. Twenty-two patients underwent contrast-enhanced CT and eight underwent non-enhanced CT. The radiologic definition of HPVG was a tubular area of low attenuation in the liver periphery, as described previously [20]. The following were transcribed from the electronic medical record: age, sex, body mass index, principal complaints, history of abdominal surgery, comorbidities, regular medication (for example, alpha-glucosidase inhibitors [9]), and the laboratory findings at admission (pH, base excess [BE], serum lactate concentration, white blood cell count, neutrophil-to-lymphocyte ratio [NLR] [21], the lactate dehydrogenase and creatine kinase activities, and the C-reactive protein [CRP] concentration). The following CT findings at diagnosis of HPVG were also reviewed: intestinal pneumatosis (IP), portomesenteric venous gas, bowel wall attenuation in the post-contrast phase, ascites, free air, type of IP [16], and the distribution of HPVG [19, 20].

**Fig. 1:**
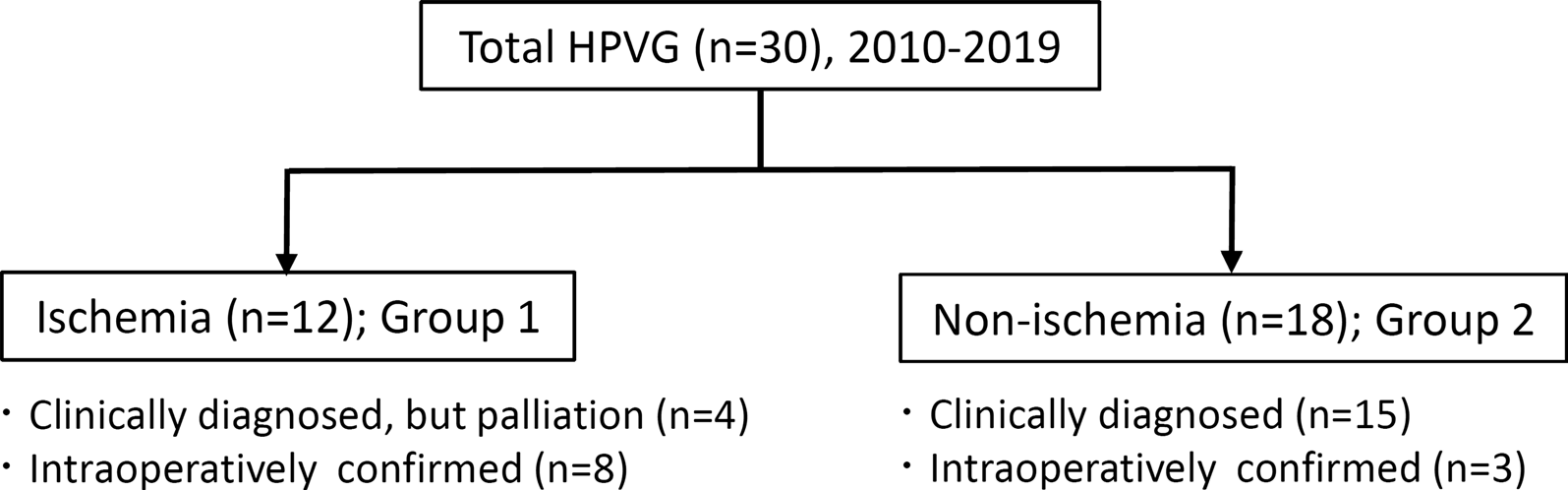
Characteristics of the study population

While clinical diagnosis of bowel ischemia was mainly obtained by enhanced CT on admission, given its well-established high sensitivity and specificity [22], comprehensive assessment based on physical, hematological, and radiological findings remained essential especially in the patients who could receive only non-enhanced CT. Emergency surgery was performed if there was a clinical suspicion of bowel ischemia. According to the intraoperative findings, an appropriate procedure (for example, exploratory laparotomy alone or bowel resection with pathologic evaluation of specimen) was performed. In terms of intraoperative bowel findings, surgeons focused on the presence or absence of return of color, arterial pulsations, and visible peristalsis. Previous literature demonstrated the favorable consistency between those gross appearances obtained by experienced surgeon and pathological results regarding the diagnosis of bowel ischemia [23]. On the basis of clinical, intraoperative, and pathologic findings, the identity of the underlying disease in the HPVG cohort was determined, then the patients were allocated to two groups: those with (Group 1; n = 12 [40%]) and those without (Group 2; n = 18 [60%]) bowel ischemia (Fig. 1). Emergency surgery was performed in 11 patients (37%) and bowel ischemia was identified in eight of these (73%). Four patients in Group 1 were clinically diagnosed with bowel ischemia but were treated palliatively because of their poor general condition (Fig. 1). We then compared the characteristics of Groups 1 and 2 to determine the potential prognostic factors for bowel ischemia. The therapeutic course after admission, including the perioperative findings, was reviewed on the patients’ medical charts.

### Statistical analyses

Continuous data were compared using the Wilcoxon rank-sum test and categorical data were compared using the χ2 test. The performance of laboratory values in the prediction of bowel ischemia was assessed using receiver operating characteristic (ROC) analysis and optimal cut-off values were determined. The accuracy with which these parameters discriminated patients with and without bowel ischemia was assessed by calculating the areas under the curves (AUCs). *P* < 0.05 was considered to represent statistical significance in all the analyses. Statistical analyses were performed using JMP software (version 15; SAS Institute, Cary, NC, USA).

## Results

### Categorization of patient characteristics according to presence of bowel ischemia

The clinical and radiologic data for patients with (Group 1; n = 12 [40%]) and without bowel ischemia (Group 2; n = 18 [60%]) are summarized in Table 1. Compared with patients in Group 2, those in Group 1 were more likely to present with the peritoneal irritation sign, to have lower BE and higher lactate, NLR, and CRP on admission. With regard to the CT findings, the incidence of a combination of IP and HPVG in Group 1was significantly higher than that in Group 2, but there were no significant differences between Groups 1 and 2 with regard to the prevalences of ascites or free air, or the distribution of HPVG. Because none of the patients had HPVG that was limited to the right side of the liver, we confirmed that the left side of the liver is the predominant site for HPVG to develop, as previously described [19, 20]. In Group 1 patients, the most common specific cause of bowel ischemia was non-occlusive mesenteric ischemia (8/12, 75%), but the four patients that only received palliative care in Group 1 were not autopsied.

**Table 1:** Patient characteristics

	Total	Ischemia (Group 1)	Non-ischemia (Group 2)	*P*^d^
	n = 30	n = 12	n = 18	
Age (years)	72 (53–92)	74 (55–90)	72 (53–92)	0.816^e^
Male, n (%)	19 (63)	6 (50)	13 (72)	0.216
Body mass index, kg/m^2^	20.9 (15.2–29.0)	22.0 (17.1–28.2)	20.6 (15.2–29.0)	0.421^e^
Principal complaints^a^				
Abdominal pain, n (%)	23 (77)	9 (75)	14 (78)	0.860
Peritoneal irritation sign, n (%)	7 (23)	7 (58)	0 (0)	< *0.001*
Nausea, n (%)	10 (33)	2 (17)	8 (44)	0.114
Impaired consciousness, n (%)	4 (13)	2 (17)	2 (11)	0.661
Gastrointestinal bleeding, n (%)	4 (13)	0 (0)	4 (22)	0.079
Abdominal fullness, n (%)	3 (10)	0 (0)	3 (17)	0.136
Fever, n (%)	2 (6.7)	1 (8.3)	1 (5.6)	0.765
History of abdominal surgery, n (%)	9 (30)	3 (25)	6 (33)	0.626
Hypertension, n (%)	18 (60)	6 (50)	12 (67)	0.361
Diabetes mellitus, n (%)	12 (40)	4 (33)	8 (44)	0.543
Use of alpha-glucosidase inhibitor, n (%)	6 (20)	1 (8.3)	5 (28)	0.192
Use of psychotropic agent, n (%)	8 (27)	3 (25)	5 (28)	0.866
Use of anticoagulant agent, n (%)	10 (33)	2 (17)	8 (44)	0.114
Use of steroid, n (%)	3 (10)	2 (17)	1 (5.6)	0.320
Laboratory findings				
pH^b^	7.42 (6.98–7.61)	7.38 (6.98–7.57)	7.42 (7.36–7.61)	0.114^e^
Base excess, mmol/L^b^	0.9 (− 27.6–6.6)	-4.4 (-27.6–2.5)	2.3 (-5.2–6.6)	*0.005*^e^
Lactate, mmol/L^b^	2.1 (0.8–13.8)	5.2 (1.2–13.8)	1.8 (0.8–5.1)	*0.003*^e^
White blood cell, /μL	9990 (2230–45,090)	12,720 (2,230–45,090)	9330 (4930–26,100)	0.290^e^
Neutrophil lymphocyte ratio^c^	9.3 (1.3–29)	21 (7.7–29)	6.6 (1.3–18)	*0.013*^e^
Lactate dehydrogenase, IU/L	207 (137–619)	207 (147–619)	207 (137–586)	0.757^e^
Creatine kinase, IU/L	68 (20–595)	71 (20–595)	60 (26–135)	0.338^e^
C-reactive protein, mg/dL	4.0 (0–48)	9.7 (1.2–48)	2.8 (0–20)	*0.016*^e^
Computed tomography findings				
Intestinal pneumatosis, n (%)	15 (50)	9 (75)	6 (33)	*0.025*
Band like pneumatosis [16], n (%)	11 (37)	8 (67)	3 (17)	*0.005*
Bubble like pneumatosis [16], n (%)	4 (13)	1 (8.3)	3 (17)	0.511
Ascites, n (%)	14 (47)	5 (42)	9 (50)	0.654
Free air, n (%)	2 (6.7)	2 (17)	0 (0)	0.073
Distribution of HPVG [19]				
Bilobar, n (%)	18 (60)	6 (50)	12 (67)	0.361
Limited to left liver, n (%)	12 (40)	6 (50)	6 (33)	0.361
Underlying disease as cause of HPVG				
Bowel ischemia	12 (40)	12 (100)	0 (0)	< *0.001*
Enteritis	10 (33)	0 (0)	10 (56)	*0.002*
Bowel distention	6 (20)	0 (0)	6 (33)	*0.025*
Pneumothorax	1 (3.3)	0 (0)	1 (5.6)	0.406
Unknown	1 (3.3)	0 (0)	1 (5.6)	0.406

Data are presented as median (range) unless otherwise indicated

Italic values indicate items represented statistical significance

*HPVG* hepatic portal venous gas

^a^Overlap of principal complaints was allowed

^b^Data of 4 patients are missing

^c^Data of 15 patients are missing

^d^χ^2^ test unless otherwise indicated

^e^Wilcoxon rank-sum test

Laboratory measurements (BE, lactate, and CRP) that were made on admission and were candidates for use in the prediction of bowel ischemia were assessed using ROC analysis (Additional file 1: Fig. S1A). This analysis was not applied to NLR because of substantial number of missing data (15/30). ROC analyses revealed that BE (AUC 0.831, *p* = 0.005), lactate (AUC 0.850, *p* = 0.003), and CRP (AUC 0.819, *p* = 0.007) were significantly associated with bowel ischemia and the optimal cut-off values for the prediction of bowel ischemia were − 5.5 mmol/L, 3.5 mmol/L, and 4.4 mg/dL, respectively (Additional file 1: Fig. S1B, C, D).

### Predictors of bowel ischemia in patients without the peritoneal irritation sign (n = 23)

**Fig. 2:**
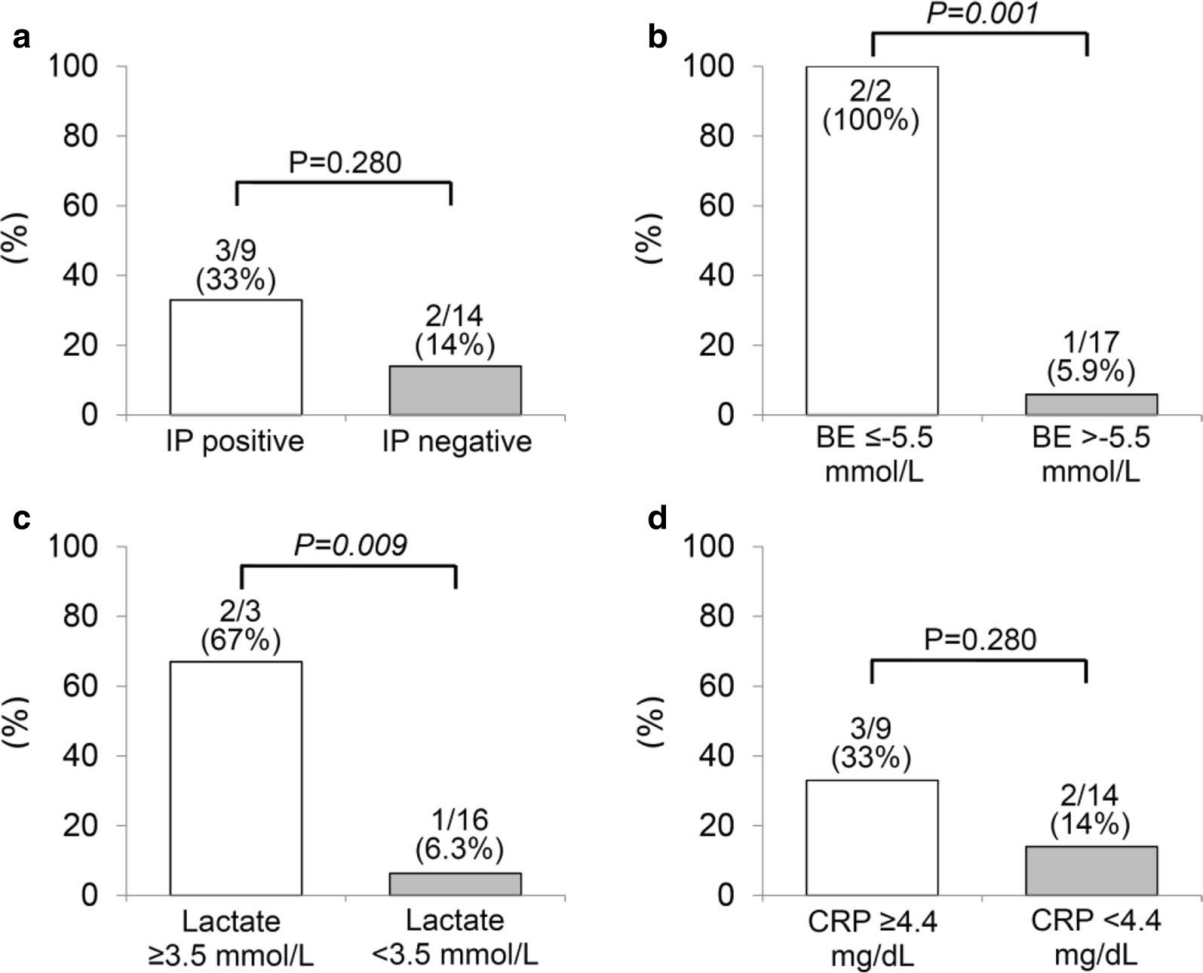
Incidence of bowel ischemia in patients without the peritoneal irritation sign (n = 23). The incidences of bowel ischemia in patients without the peritoneal irritation sign were compared using the χ^2^ test. IP, intestinal pneumatosis; BE, base excess; CRP, C-reactive protein

On the basis of the data in Table 1, emergency surgery is clearly justified in HPVG patients with the peritoneal irritation sign, because of its 100% (7/7) positive predictive value for bowel ischemia. However, it may be more important to identify the presence of bowel ischemia in HPVG patients without the peritoneal irritation sign, because its negative predictive value for bowel ischemia was unsatisfactory (18/23, 78%) (Table 1). Therefore, we analyzed the incidence of bowel ischemia in patients without the peritoneal irritation sign (n = 23), focusing on IP on CT finding, BE, lactate, and CRP (Fig. 2). To dichotomize BE, lactate, and CRP, accurate thresholds were selected according to the results of the ROC analyses (Additional file 1: Figure S1). Lower BE and higher lactate on admission remained significant predictors of bowel ischemia, even in HPVG patients without the peritoneal irritation sign (Fig. 2).

### Therapeutic course

**Fig. 3:**
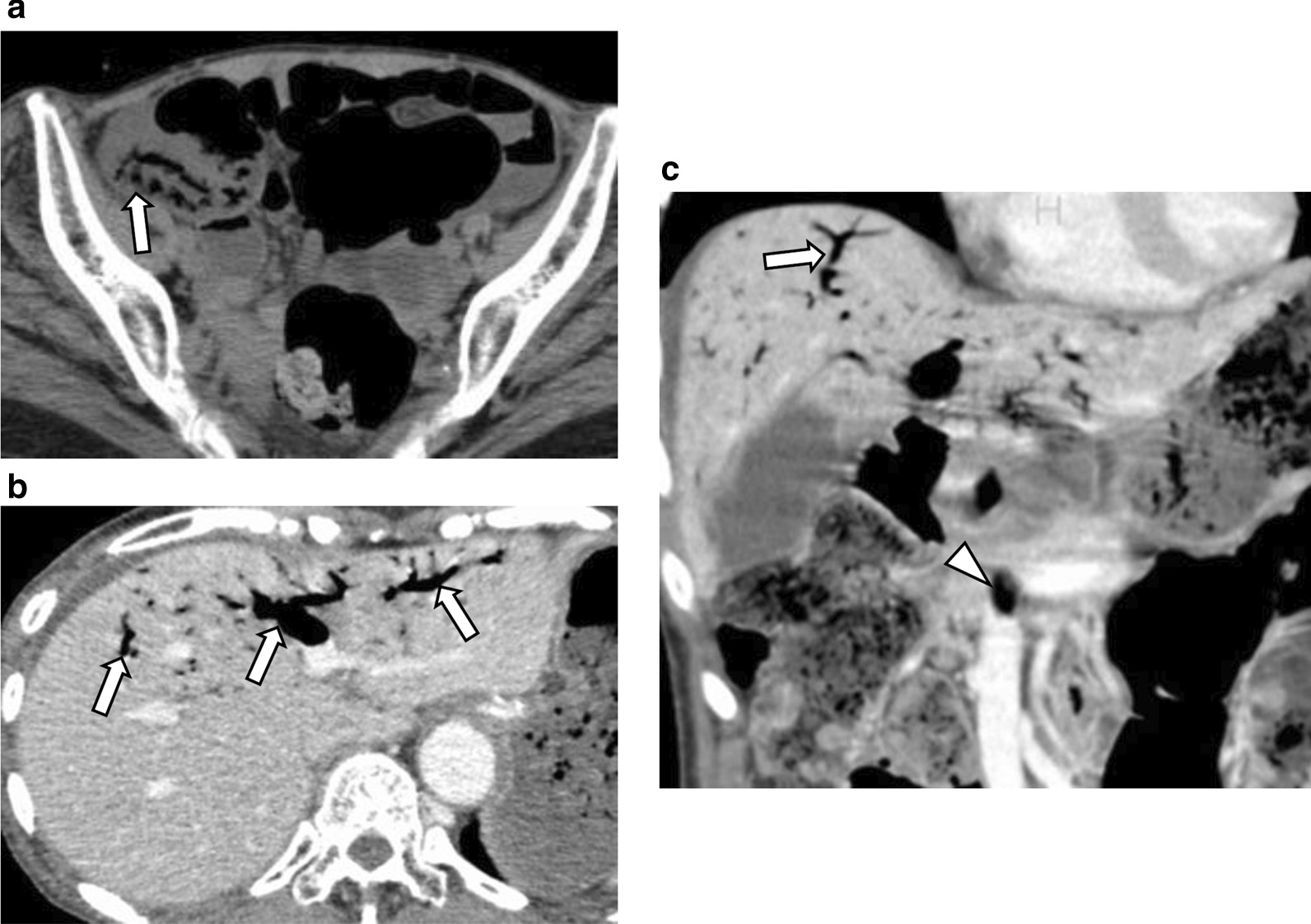
Representative case with hepatic portal venous gas. A 70–80-year-old thin patient, who had experienced multiple system atrophy, associated with poor Eastern Cooperative Oncology Group performance status, presented to the emergency department complaining of abdominal distension and nausea. An abdominal CT performed on admission revealed diffuse distension of the small intestine, a suspicion of pneumatosis in the terminal ileum (**a** arrow), and portal (**b**, **c** arrow) and mesenteric venous gas (C, arrowhead). Given the absence of solid evidence of bowel obstruction and ischemia (lack of the peritoneal irrigation sign, a base excess of − 1.6 mmol/L, and a lactate concentration of 2.7 mmol/L), it was decided to treat her with fasting and antibiotics. Following radiological confirmation of significant improvement in the ileal pneumatosis and portal and mesenteric venous gas on the third day following admission, she was discharged in a satisfactory condition after 4 weeks of conservative therapy

Table 2 summarizes the perioperative findings in Group 1 patients, particularly those who underwent emergency surgery (n = 8). Although emergency surgery was performed after a median of only 7.1 h post-arrival, the postoperative outcomes were still poor (50% mortality [4/8]). Of note, three of the four patients who underwent bowel resection and primary anastomosis experienced anastomotic leak, which lead to mortality in all three cases. The cause of death of the remaining patient, who had undergone successful adhesiolysis, was severe sepsis due to preoperative aspiration pneumonia. Four patients who showed clinical evidence of bowel ischemia but underwent palliation alone died a median of 1 day following admission (range, 1–6 days). In general, patients in Group 2 showed satisfactory therapeutic courses when undergoing conservative therapy alone, depending on the identity of the underlying disease, with a median of 16 days of hospitalization (range 3–45), except for one who died of aspiration pneumonia secondary to enteritis on the 14th day following admission. Images of a representative case of HPVG are shown in Fig. 3. The mortality rate for the full cohort that had presented with HPVG during the past decade was 30% (9/30).

**Table 2:** Operative findings in patients undergoing surgery for ischemia (n = 8)

Interval from arrival to surgery, median (range), h	7.1 (3.4–34)
Surgical procedure	
Small bowel resection, n (%)^a^	4 (50)
Colectomy, n (%)^b^	2 (25)
Primary repair for perforation, n (%)	1 (13)
Adhesiolysis, n (%)	1 (13)
Operative time, median (range), min	148 (45–198)
Estimated blood loss, median (range), cc	85 (10–330)
Mortality, n (%)	4 (50)
Postoperative survival in mortality cases, median (range), days	8.5 (2–59)

^a^Three of 4 patients underwent primary anastomosis and the other had prophylactic stoma

^b^One patient underwent primary anastomosis and the other had prophylactic stoma

## Discussion

This study found that while the majority of HPVG patients (18/30, 60%) did not have bowel ischemia, it was the most common specific underlying disease in adults with HPVG (12/30, 40%), which is consistent with earlier reports [3, 18]. Our results suggested that bowel ischemia in HPVG patients should still be regarded as an ominous radiologic sign, in contrast to the favorable outcomes in HPVG patients without bowel ischemia. Indeed, the mortality of HPVG patients with bowel ischemia who had undergone surgery was 50% (4/8). In particular, three out of four patients who underwent bowel resection and primary anastomosis suffered from anastomotic leak, which directly lead to mortality, suggesting that the use of an earlier second-look laparotomy and/or a prophylactic stoma should be considered in such patients. Modern surgical techniques, such as fluorescent angiography for the verification of intraoperative vascular perfusion, might have a positive effect on the prognosis of those who are predisposed to postoperative anastomotic leak [24].

We also found that the peritoneal irritation sign, several laboratory findings on admission (BE, lactate, NLR, and CRP), and IP on CT represented potential prognostic factors for bowel ischemia in the full cohort with HPVG, although this finding was based on univariable analyses, because of the relatively small sample size. In particular, dichotomized BE and lactate remained significant predictors of bowel ischemia, even in HPVG patients without the peritoneal irritation sign (n = 23). Patients who present with obscure symptoms, because of their highly advanced age or impaired consciousness, for example, might benefit from measurement of these variables at diagnosis. These variables, other than NLR, have been already reported to be candidate predictors of bowel ischemia in patients with HPVG [16, 25–30]. However, no previous reports have described an association between high NLR and bowel ischemia in HPVG patients, although some researchers have suggested that NLR might be useful for the identification of, and assessment of the severity of, acute mesenteric ischemia [31]. Further research on nomogram development integrating such potentially predictable factors on bowel ischemia may be required for swifter determination of operative indication in HPVG patients, given the current dismal mortality rate in patients with bowel ischemia undergoing emergency surgery.

In the present cohort, three of eleven patients (27%) undergoing emergency surgery had an exploratory laparotomy alone, due to intraoperative recognition of the absence of bowel ischemia, and were subsequently managed without bowel resection. Such over-diagnosis might be justified in the context of the highly unfavorable outcomes in HPVG patients who do have bowel ischemia. However, further refinement of the methods used for the identification of underlying disease in HPVG patients should reduce the need for unnecessary surgery. This might also contribute to more rapid decision-making regarding the necessity for emergency surgery, which could reduce mortality, although in the present study there was a median of only 7.1 h (range 3.4–34) between arrival and surgery. In addition, the identification, not only of the presence of bowel ischemia in HPVG patients, but also of its severity, would be clinically relevant for the selection of the safest procedure (for example, the creation of a prophylactic stoma or not) during emergency surgery. For instance, previous studies have demonstrated that the type of IP (band-like or bubble-like) is significantly associated with the severity of bowel ischemia [16, 32], but this relationship could not be validated in the present, relatively small cohort.

The present study had a few limitations, including its retrospective nature and the small number of patients with HPVG studied, which meant that multivariable analysis of the potential predictors of bowel ischemia could not be performed. Second, there was a significant proportion of patients (4/30, 13%) who presented with impaired consciousness as their principal complaint, and whose physical findings, including the peritoneal irritation sign, were difficult to establish. Third, eight of the 30 patients (27%) were diagnosed using non-enhanced CT, because of renal functional impairment or drug allergy. Moreover, in Group 1 (n = 12), diagnosis of bowel ischemia was made by only clinical findings in 4 patients and the remaining 8 patients obtained intraoperative confirmation of bowel ischemia. But such diagnostic heterogeneity is reflective of general clinical practice and is consistent with previous findings [16].

## Conclusions

While HPVG can be the result of a wide spectrum of underlying diseases, including non-ischemic conditions, critical abdominal pathologies should be identified promptly and accurately, given the high mortality in patients with bowel ischemia even undergoing emergency surgery. Further advancements in diagnostic capabilities, with the use of parameters that are potentially predictive of bowel ischemia, and in perioperative management, including the selection of safer surgical procedures, might improve the outcomes in HPVG patients.

## Supplementary information

**Additional file 1: Figure S1.** Receiver operating characteristic curves for the use of laboratory parameters measured at admission for the prediction of bowel ischemia. The accuracy for the discrimination of patients with and without bowel ischemia was assessed by calculating the areas under the curves for base excess, lactate, and C-reactive protein on admission (A). Appropriate thresholds for the prediction of bowel ischemia were determined to be − 5.5 mmol/L for base excess (B), 3.5 mmol/L for lactate (C), and 4.4 mg/dL for C-reactive protein (D). AUC, area under the curve; CI, confidence interval; SE, standard error. The *P* values indicate the usefulness of the parameter as a predictor (null hypothesis, AUC = 0.500).

## Data Availability

All data and materials concerning this research article are available for sharing if needed.

## Abbreviations

HPVG: Hepatic portal venous gas
CT: Computed tomography
BE: Base excess
NLR: Neutrophil-to-lymphocyte ratio
CRP: C-reactive protein
IP: Intestinal pneumatosis
ROC: Receiver operating characteristic
AUCs: Areas under the curves
