# Supplementary figures and images for: Clinical features of patients with hepatic portal venous gas
Source: BMC Surg. 2020 Nov 27;20:300. doi: 10.1186/s12893-020-00973-8 (PMC7694268; doi:10.1186/s12893-020-00973-8)

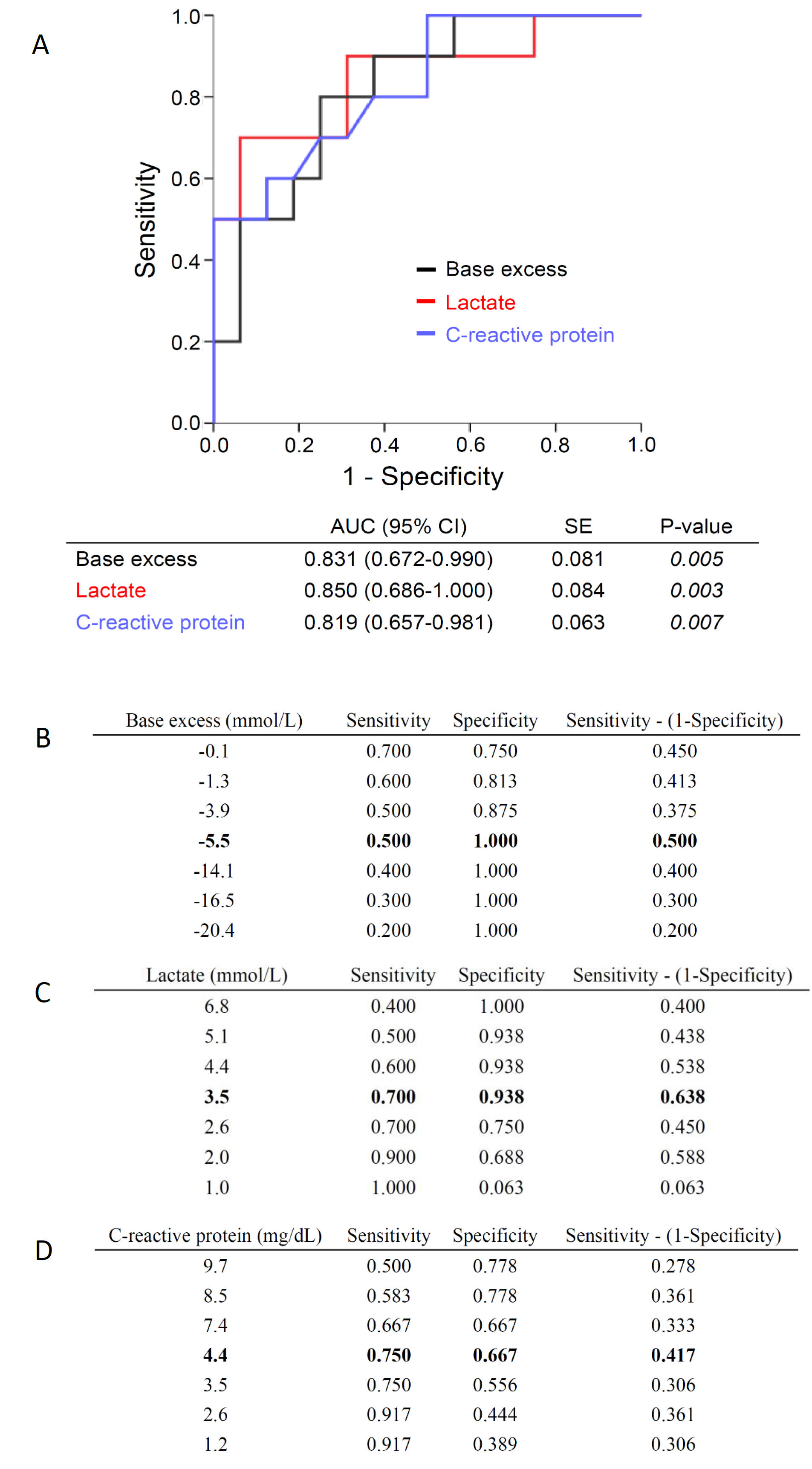

Supplement: Supplementary file 1 — Additional file 1: Figure S1. Receiver operating characteristic curves for the use of laboratory parameters measured at admission for the prediction of bowel ischemia. The accuracy for the discrimination of patients with and without bowel ischemia was assessed by calculating the areas under the curves for base excess, lactate, and C-reactive protein on admission (A). Appropriate thresholds for the prediction of bowel ischemia were determined to be − 5.5 mmol/L for base excess (B), 3.5 mmol/L for lactate (C), and 4.4 mg/dL for C-reactive protein (D). AUC, area under the curve; CI, confidence interval; SE, standard error. The P values indicate the usefulness of the parameter as a predictor (null hypothesis, AUC = 0.500). [file 12893_2020_973_MOESM1_ESM.tif]
